# Design and Finite Element Analysis of Patient-Specific Total Temporomandibular Joint Implants

**DOI:** 10.3390/ma15124342

**Published:** 2022-06-20

**Authors:** Shirish M. Ingawale, Tarun Goswami

**Affiliations:** 1 Department of Biomedical, Industrial & Human Factors Engineering, Wright State University, 3640 Col Glen Hwy, Dayton, OH 45435, USA; shirishingawale@gmail.com; 2 Department of Orthopaedic Surgery and Sports Medicine, Wright State University, Dayton, OH 45435, USA

**Keywords:** TMJ, biomaterials, custom devices, finite element analysis, 3D models, mandibles

## Abstract

In this manuscript, we discuss our approach to developing novel patient-specific total TMJ prostheses. Our unique patient-fitted designs based on medical images of the patient’s TMJ offer accurate anatomical fit, and better fixation to host bone. Special features of the prostheses have potential to offer improved osseo-integration and durability of the devices. The design process is based on surgeon’s requirements, feedback, and pre-surgical planning to ensure anatomically accurate and clinically viable device design. We use the validated methodology of FE modeling and analysis to evaluate the device design by investigating stress and strain profiles under functional/normal and para-functional/worst-case TMJ loading scenarios.

## 1. Introduction

In treating the TMJ dysfunction, all nonsurgical approaches should be exhausted. In some select patients, the end-stage TMJ pathology resulting in distortion of anatomical architectural form and physiological dysfunction dictates the need for total joint replacement (TJR) [1,2,3,4]. The goal of TMJ TJR is the restoration of mandibular function and form; any pain relief attained is considered of secondary benefit [1,2]. The TMD patients with serious osteoarthritis, rheumatoid arthritis, psoriatic arthritis, and ankylosis might be good candidates for receiving TMJ prosthesis [1,2,3,4,5,6,7,8].

TMJ resections have been carried out for about 150 years [4,9,10]. Before 1945, the technique of alloplastic reconstruction of TMJ was mainly limited to replacement of condyle [9]. Interposition of alloplastic implants, resection dressings and prostheses were the dominant techniques [9]. Sterilization, biocompatibility and fixation of the alloplastic implants were main concerns in early days [9]. No evidence-based data on outcomes are available from that time. By 1945 reconstruction of the TMJ involved the close cooperation of surgeons and dentists [5,9]. In view of the rare application of TMJ prostheses, their relatively wide variety described over past six decades emphasizes that alloplastic TMJ reconstruction is still evolving.

TMJ implants can be differentiated into fossa-eminence prostheses, ramus prostheses and condylar reconstruction plates, and total joint prostheses. Although singular replacement of the fossa or condyle is preferred as a temporary solution, the partial TMJ reconstruction finds comparatively declining usage by surgeons for clinical reasons. Total TMJ implants are recommended when the glenoid fossa is exposed due to excessive stress in conditions such as degenerative disorders, arthritis ankylosis, and multiply operated pain patients [1,2,3,4,5,6,7]. Table 1 lists indications for alloplastic reconstruction of the TMJ.

Relative contraindications to the use of alloplast in reconstruction of the TMJ are age of the patient, mental status of the patient, uncontrolled systemic disease such as diabetes mellitus or myelodysplasia, active or chronic infection at the implantation site, and allergy to materials that are used in the devices to be implanted [1,3,4]. The perceived potential disadvantages of the alloplastic TMJ TJR are cost of the device, need for two-stage procedure in ankylosis cases, material wear debris with associated pathologic responses failure of the prostheses secondary to loosening of the screw fixation or fracture from metal fatigue, lack of long-term stability, inability of alloplastic implant to follow physical growth of the younger patients, and unpredictable need for revision surgery. Long-term studies comparing functional and aesthetic outcomes of various TMJ prostheses are not available (with an exception of one study by [11] with up to 14-year follow-up), which leaves the choice of prosthesis to surgeon’s personal preference.

We performed a comprehensive review of published literature [1,2,3,4,5,6,7,8,9,10,11,12,13,14,15,16,17,18,19,20,21,22,23,24,25,26,27,28,29,30,31,32,33,34,35,36] regarding TMJ reconstruction, and based our TMJ prostheses design approach on the knowledge gained from clinical, biomechanical and scientific reports about the history, designs, efficacy, and clinical outcomes of TMJ prostheses. There are two categories of the TMJ TJR devices approved for implantation by the United States Food and Drug Administration (FDA); the stock or off-the-shelf devices, and the custom or patient-fitted devices. At the time of implantation, the surgeon has to ‘make fit’ the stock (off-the-shelf) device. In contrast, the custom (patient-fitted) devices are ‘made to fit’ each specific case. To date, there is only one study [13], reported in the literature that compares a stock and a custom TMJ TJR system. This study concluded that patients implanted with the custom TMJ TJR system had statistically significant better outcomes in both subjective and objective domains than did those implanted with the stock system devices studied [13].

The history of alloplastic TMJ reconstruction has, unfortunately, been characterized by multiple highly publicized failures based on inappropriate design, lack of attention to biomechanical principles, and ignorance of what already had been documented in the orthopedic literature [3,4,12,14]. In addition, because TMJ is the only ginglymoarthrodial joint in human body, and because its function is intimately related to occlusal harmony, a prosthetic TMJ necessitates characteristics not considered in orthopedic implant design [4]. The use of inappropriate materials and designs has resulted in success rate of many TMJ implants being lower than those for total hip and knee prostheses [14]. Most of the published literature regarding TMJ implants has been clinical and case reports, with much less studies investigating the design and biomechanics of the TMJ implants. In view of paucity of this information, and the need for more efficient and durable total TMJ implants, we undertook a study aimed at designing and evaluating customized total TMJ prostheses.

### Design Requirements for Total TMJ Prosthesis

van Loon, et al. [15] indicated that there are three major requirements in TMJ TJR; (i) to imitate the functional movement, (ii) to realize a close fit to the skull, and (iii) to achieve a long lifetime. Table 2 lists summary of requirements for successful total reconstruction of the TMJ. Stability of alloplastic joint replacements depends not only on fixation, but also on adaptation of the implant to the bone to which it is to be fixed [1,2,11]. The orthopedic experience with implantation of alloplastic joints has shown that better adaptation of the device to the host bone results in more stability and functional longevity of the implant [1,2,16,17]. Stability of TMJ prosthesis at the time of implantation is equally important for its success. Motion of the implanted prosthesis under a load can cause the surrounding bone to degenerate, leading to further device loosening and consequent failure [1]. Currently, screw fixation of TMJ implants is the most predictable and stable form of stabilization developed [1]. Screws may loosen with time and function, requiring replacement. To assure long-term success of the TMJ implants, primary stability of prosthetic components must be ensured by biointegration of the screws [2].

Most patients requiring TMJ replacement have deformed local bony anatomy. During implantation of the stock TMJ prosthesis, the surgeon confronts with a difficult challenge of making ‘off-the-shelf’ components fit and remain stable, and often the precious host bone needs to be sacrificed to make the stock TMJ components to create stable component-to-host-bone contact [2]. Surgeons attempt to make stock devices fit by bending or shimming may lead to component or shim material fatigue and/or overload fostering early failure under repeated cyclic functional loading. Potential micromotion of any altered or shimmed component adversely affects the screw fixation biointegration. Micromotion leads to the formation of a fibrous connective tissue interface between the altered component and the host bone, and can cause early loosening of the screws leading to device failure. Our patient-specific TMJ implants are designed to accurately fit each patient’s specific anatomical condition. They conform to any unique or complex anatomical host bone condition. These designs do not require any alteration or shimming of either the device or the host bone to achieve initial fixation and stability. The screws secure implant components intimately to the host bone mitigating possibility of micromotion and maximizing the opportunity for biointegration [2].

## 2. Materials and Methods

In this section, we discuss the methodology of designing condylar and fossa components of the custom-designed total TMJ prostheses. Also discussed, are unique design features such as accurate fit of the prosthetic surface to the host bone in contact, perforated notches of implant which protrude and fit into the custom-cut slots in native bone, customized surgical guides, and screws with locking mechanism.

### 2.1. Design of Patient-Specific Total TMJ Prosthesis

**Figure 1:**
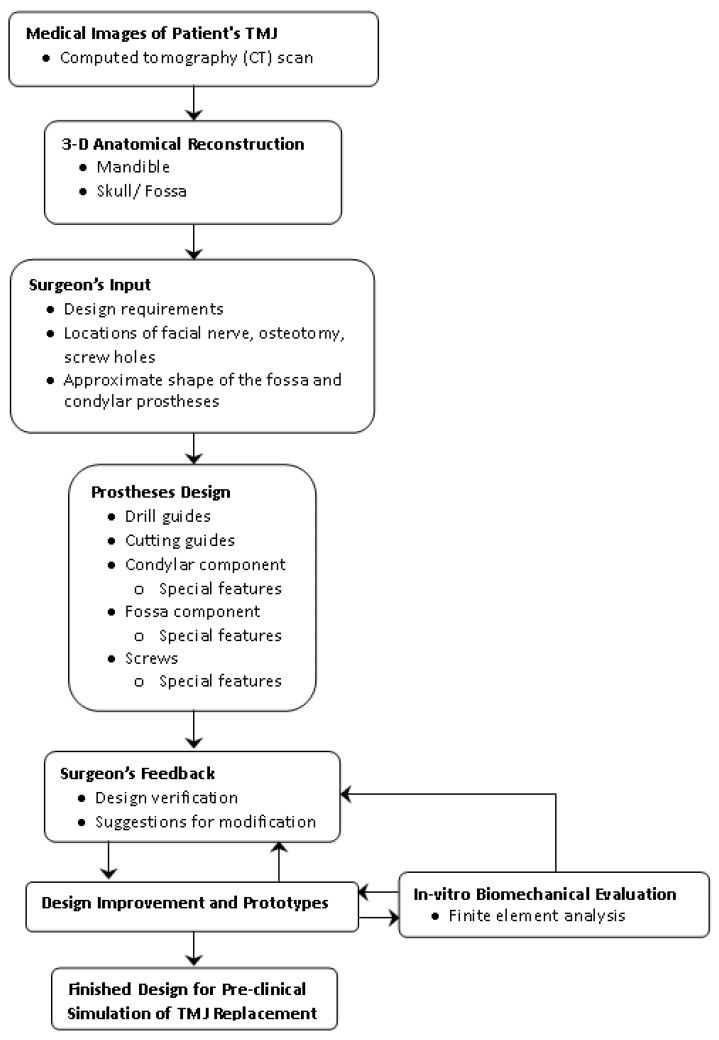
Methodology followed for design and preliminary analysis of the patient-specific total temporomandibular joint (TMJ) prostheses.

The schematic in Figure 1 outlines our approach to developing a novel patient-specific total TMJ implant system. Our unique patient-fitted designs based on computed tomography (CT) images of the patient’s TMJ and associated anatomic structures offer accurate anatomical fit and better fixation to the host bone. The novel/unique features of the prostheses promise an improved osseo-integration and durability. Our design process is based on surgeon’s requirements, feedback, and pre-surgical planning to ensure anatomically accurate and clinically viable device design. Pre-planning of the surgery is an integral part of the proposed design and development methodology, and is intended to reduce intra-operative adjustments of the device components, complexity of the already challenging operating procedure, and the overall time spent in the operating room.

**Figure 2:**
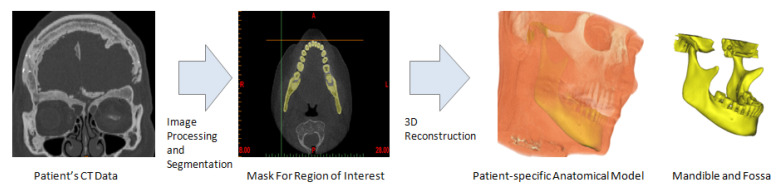
Subject-specific 3D anatomical reconstruction of the patient’s mandible and fossa eminence performed from computed tomography (CT) data using Mimics software.

Subject-specific 3D anatomical reconstruction of the patient’s mandible and skull/fossa/articular eminence is performed using commercial software Mimics v14.12 (Materialise, Plymouth, MI, USA) from computed tomography (CT) scans (see Figure 2). Upon importing the patient’s CT images in Mimics, anatomical model comprising of the patient’s mandible and fossa eminence is developed by performing a series of operations such as image processing, segmentation, region growing, mask formation for the anatomic region of interest (i.e., bone and teeth), and calculation of 3D equivalent similar to the 3D reconstruction method described elsewhere [18]. The prostheses and accessories are designed using commercial software packages 3-matic v6.0 (Materialise, Plymouth, MI, USA) and SolidWorks v2010 (SIMULIA, Providence, RI, USA) as discussed in following sections.

#### 2.1.1. Surgical Pre-Planning and Surgeon Input

Close collaboration between device designer and surgeon (treating the given TMJ patient) is an important aspect of the proposed design and development approach. Mutual sharing of knowledge and expertise, clinical and design requirements and constraints is vital in ensuring the optimal design and performance of TMJ devices. We utilize the computerized anatomical model and its 3D printed equivalent to acquire surgeon’s design requirements such as location of the facial nerve (to keep it from any damage or injury during surgery), location of the condylar osteotomy (i.e., removal of the degenerated or damaged condylar bone), outline of shape for the planned condylar and fossa prostheses, location of screws to secure the condylar and fossa components to host bone, number and dimension of screws, etc.

**Figure 3:**
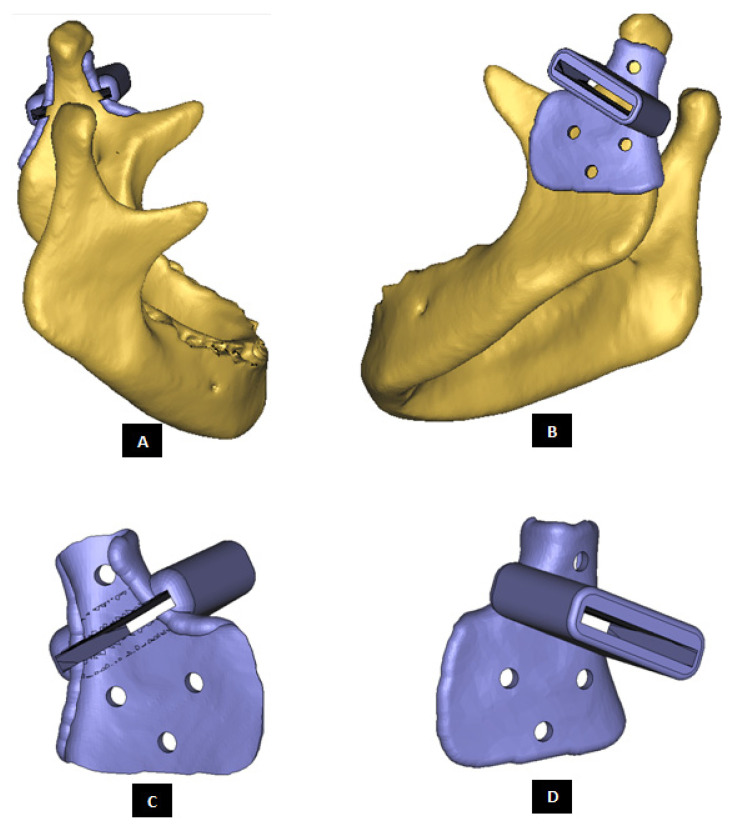
Custom-designed surgical guide for condylectomy (i.e., removal of damaged part of the condylar bone). (**A**,**B**) Show the medial and lateral view, respectively, of surgical guide placed at the location on mandible where osteotomy is to be performed. (**C**,**D**) Show medial and lateral–anterior view, respectively, of the surgical guide alone. The visuals demonstrate that custom-design of the device enables it to accurately adapt to the native bone. This methodology allows the designer to control size and shape of the device, and location of its fixation screws as prescribed by the surgeon.

To help the surgeon accurately remove the damaged part of condylar neck/head, a surgical guide is custom designed for each reconstruction case as shown in Figure 3. During surgery, after putting the patient in intermaxillary fixation (IMF) and gaining access to TMJ capsule, the surgical guide can be fixated to mandible using screws located superior and inferior to the line of osteotomy/condylectomy. In other words, the surgical guide is secured using screws at the condylar head and condylar neck/ramus depending on osteotomy location and surgeon’s preference. After completing the osteotomy, surgical guide is detached from the bone by removing the screws. The location of condylectomy guide screw hole inferior to the anterio-posterior excision line can be selected (and custom designed) such that the same screw hole can also be used later by one of the screws used to secure condylar/ramus implant to the host mandible.

Our design approach and pre-surgical planning enables appropriate design of screws and pre-drilled screw holes in implants to avoid unintentional injury to facial nerve, soft tissue, and other delicate structures in the vicinity of the complex surgical site. Following the similar design approach used for osteotomy guide, the screw-drill-guide is custom designed each for the condylar/ramus component and the fossa-eminence part of the TMJ prostheses. These drill guides are intended to create a hole of preferred dimension (diameter and depth) at the accurate location and orientation for each screw as prescribed by the surgeon. For a given screw, a drill of smaller diameter than that of the particular screw is selected so ensure less bone damage, optimal purchase, and rigid interface between the screw and host bone during and after implantation.

Based on a surgeon’s initial design requirements, the prostheses, drill guides, osteotomy guide, and templates are designed. In response to surgeon’s feedback about the initial designs, suggested changes are incorporated to improve the device design. This feedback loop is kept open, and the designs are fine-tuned, till the surgeon approves the designs. In-vitro biomechanical assessment of patient’s host bone and TMJ prostheses is incorporated in the design validation and improvement loop as described later in this paper. After sufficiently improving the designs, the prostheses graduate to the next stage where finished implants and accessories are ready for prototyping and pre-surgical simulation of the operating procedure using anatomical models and finished prototypes. In real-world scenario, before applying our methodology to actual clinical application, it has to be verified and validated through cadaver studies.

#### 2.1.2. Design of Condylar Prosthesis

**Figure 4:**
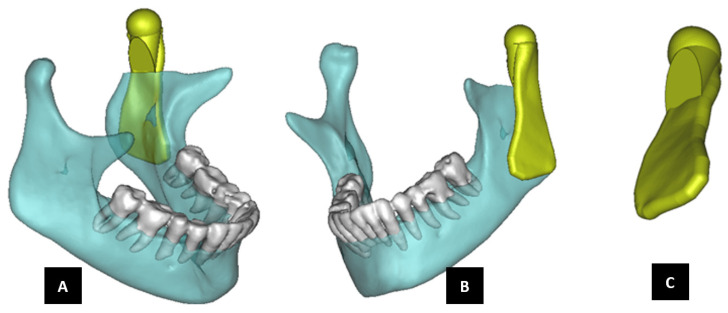
Shape outline of a custom-designed condylar/ramus prosthesis. (**A**,**B**) Show medial–anterior view and lateral–anterior view, respectively, of the prosthesis accurately adapting to the host bone. (**C**) Shows medial-inferior view of the prosthesis shape outline.

**Figure 5:**
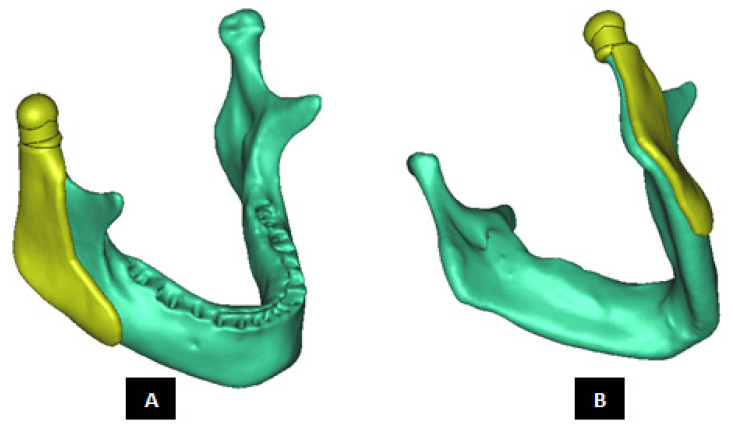
Shape outline of a custom-designed condylar/ramus prosthesis for the replacement of right TMJ of a patient. (**A**,**B**) Show lateral–anterior view and lateral–posterior view, respectively, of the prosthesis accurately conforming to geometric shape of patient’s mandible.

**Figure 6:**
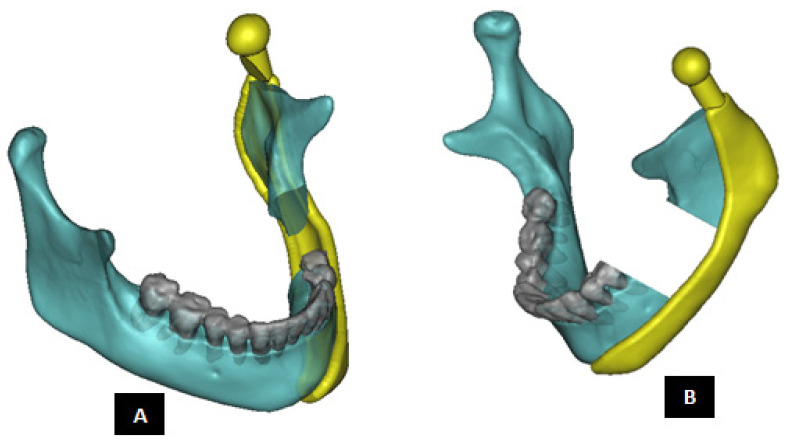
Shape outline of a custom-designed condylar/ramus/mandibular component of the TMJ prosthesis for reconstruction of left TMJ. (**A**,**B**) Show medial–anterior view and lateral–anterior view, respectively, of the prosthesis along with 3D anatomical model of the patient’s mandible after condylectomy. The osteotomy gap seen in the left mandibular body is due to removal of a tumor in that region. This osteotomy gap can be filled with a graft, and the mandibular component of this TMJ prosthesis is designed to provide mechanical support to the host bone and graft.

**Figure 7:**
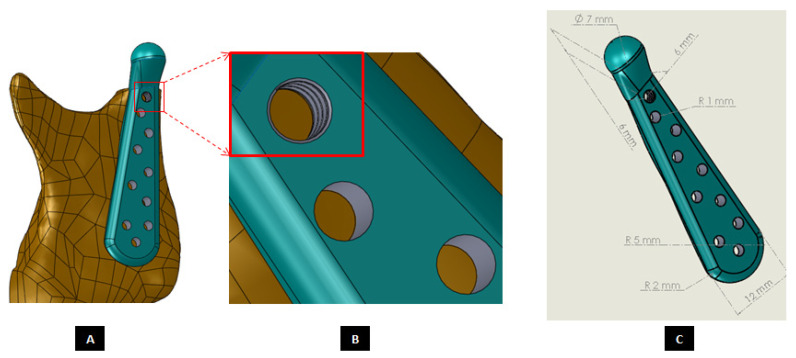
Custom-designed condylar/ramus component of the TMJ total joint replacement prosthesis for left TMJ of a patient. (**A**) Shows lateral view of the implant with screw holes. (**B**) Shows an enlarged view of the screw holes, where the first superiorly located screw hole has threads to incorporate locking-plate-screw mechanism by engaging the threads on the head of a locking screw described in the text. (**C**) Shows engineering dimensions of this patient-specific implant.

**Figure 8:**
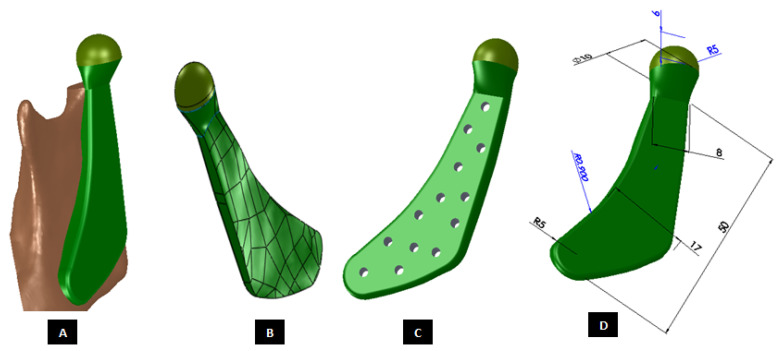
Shape outline of a custom-designed condylar/ramus component of the TMJ total joint replacement prosthesis for left TMJ of a patient. (**A**) Shows anterior–lateral view of the implant with host bone after condylectomy. The posterior–medial view in (**B**) shows that the medial surface of prosthesis is shaped to accurately follow geometric contours of the lateral surface of mandibular host bone for optimal geometrical match between the implant and host bone. (**C**) Shows lateral view of the prosthesis with screw holes. Dimensions of various parts of this patient-specific implant are shown in (**D**).

**Figure 9:**
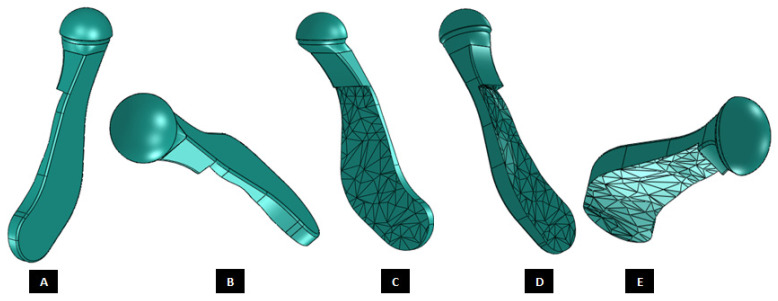
Shape outline of a custom-designed condylar/ramus component of the TMJ total joint replacement prosthesis for left TMJ of a patient. Visuals in (**A**–**E**) demonstrate that shape of medial surface of the prosthesis accurately follows the geometric contours of the lateral surface of the mandibular host bone, and maximizes the opportunity for optimal adaptation of implant to the host bone. The lateral surface of the implant is flat, condylar head is spherical, and the condylar neck has a curvature to avoid problems seen in most right-angled designs of orthopaedic implants.

**Figure 10:**
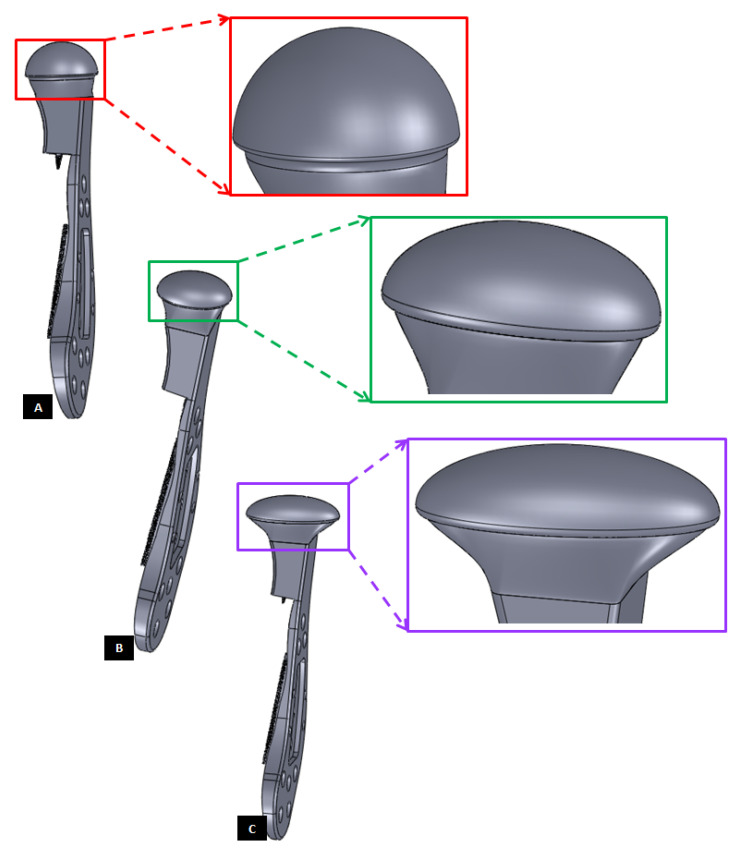
Different shapes of the condylar head of the custom-designed condylar/ramus component of the TMJ prosthesis. (**A**) Shows a prosthesis with spherical condylar head. (**B**,**C**) Show prostheses with elliptical head of different dimensions. The condylar heads are designed to offer larger articulating surface area to avoid stress concentration at small area which may lead to more wear of the articulating surfaces of reconstructed TMJ.

**Figure 11:**
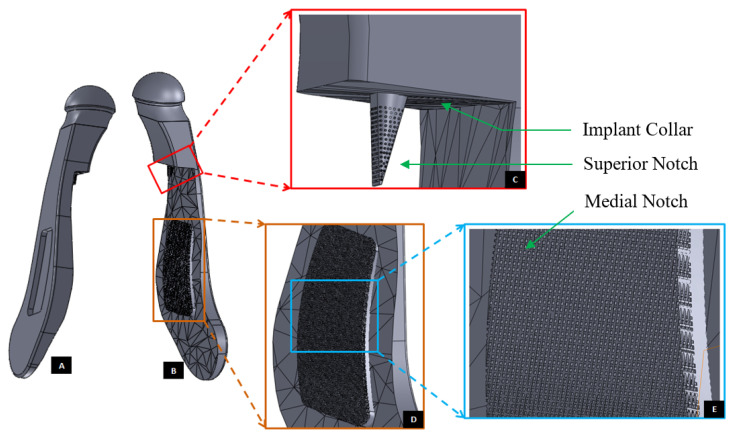
Modification of the custom-designed condylar/ramus component, shown in Figure 9, to include a novel feature; perforated notches protruding into host bone at implantation. (**A**) Shows a grove in the flat lateral surface of the condylar implant. The opposite side of this grove, as shown in (**B**), protrudes out of the medial surface as a notch with perforations. The enlarged views of medial notch and its perforations are shown in (**D**,**E**). The device also has pointed and perforated notch protruding from inferior surface of the implant’s collar/neck. Perforated surfaces of these notches are designed to permit bone in-growth into the prosthesis after implantation to provide added stability. Dimensions of these notches can be customized to fit the size and shape of patient’s native bone. Protrudes out of the medial surface as a notch with perforations (**C**).

**Figure 12:**
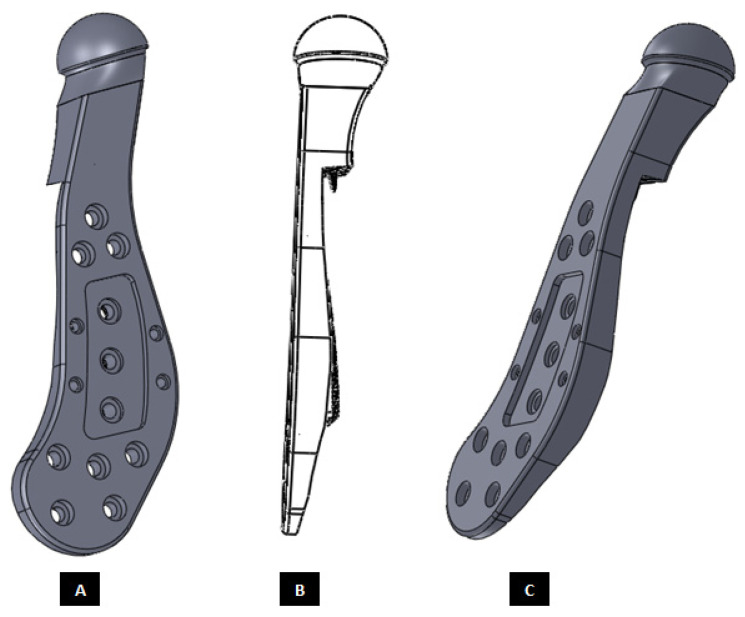
Modification and refinement of custom-designed condylar/ramus component shown in Figure 9 and Figure 11. (**A**,**C**) Show pre-drilled screw holes and a grove in the lateral surface of implant. As shown in (**C**), lateral surface of the device is flat and medial surface is shaped to match the host bone geometry. (**B**) Shows a perforated notch each protruding from the medial surface of the ramus and inferior surface of the implant collar/neck.

Anatomically accurate fit of TMJ prosthesis to the host bone is essential for stable fixation leading to efficacy and longevity of the device. Our custom-designed condylar components follow the anatomical geometry and contours on the lateral surface of ramus and condylar part of host anatomy to which the prosthesis is to be fixated. Custom shape of the prosthesis maximizes the possibility of precise fit and secure fixation. Different shapes of the condylar and ramal parts can be designed per surgeon’s recommendations to conform to the patient’s unique/complex anatomical situation. Figure 4, Figure 5, Figure 6, Figure 7, Figure 8, Figure 9, Figure 10, Figure 11 and Figure 12 show various such shapes of the condylar component of our TMJ prostheses. Since these components are custom designed to meet the unique requirements of each individual patient’s situation, the characteristic length, width, and thickness of condylar component; the number and locations of screws; and dimensions of condylar neck and head vary from patient to patient. The minimal level of the condylar thickness, width, head diameter, and number and location of screws are maintained (based on orthopaedic experience listed in the literature, surgeon’s prescription, and pre-clinical biomechanical evaluation of the device designs) to ensure that the device provides sufficient mechanical strength and stability during functional and para-functional loading after implantation.

An important advantage of our patient-specific design approach is that the components can be precisely designed to withstand the loads encountered by unique anatomic condition. For the custom-designed condylar/ramus component, the center of rotation of its head can be moved vertically to correct the open bite deformity. The prosthetic condylar head can be placed such that its center of location in located inferior to that of the natural condyle it replaced, thereby allowing low-wear articulation of the reconstructed total TMJ and natural movements of the non-replaced contra-lateral TMJ. Ramal component can be shaped to accommodate the amount of available mandibular host bone. The condylar heads can be designed in different shapes to offer larger articulating surface area to avoid stress concentration in small area of the articulating condylar head and fossa as illustrated in Figure 10. Figure 4, Figure 5, Figure 6, Figure 7, Figure 8, Figure 9, Figure 10, Figure 11 and Figure 12 show custom-designed condylar/ramus prostheses of varying shape and size. These models demonstrate that our methodology of custom design enables the condylar component to conform to the anatomic situation of damaged and/or complex mandibular host bone. Though the shown designs of condylar component vary in size and shape per anatomic demands and surgeon’s prescription, an important common design feature among all these models is that each device provides accurate adaptation to the host bone.

One novel feature of our TMJ prostheses is the perforated notches protruding into the host bone. Figure 11 and Figure 12 show a condylar/ramus component with its medial surface accurately following the geometric shape of patient’s mandibular bone. Also seen protruding out of the medial surface of this device is a rectangular notch with perforations on its surface. This notch is intended to be placed in a custom-cut grove to be created on the lateral surface of mandibular ramus by the surgeon during implantation. Custom-designed cutting guides and templates can be provided to the surgeon to accurately create a small grove in the host bone. This intentional removal of native bone is performed in exchange of the opportunity for maximizing implant stability through bony ingrowth into perforated surfaces of the notch. Figure 11 and Figure 12 show a perforated notch protruding from the inferior surface or collar of condylar neck. This notch is intended to be placed into a custom-cut grove in the superior surface of mandibular condyle/ramus resulting from osteotomy (performed to remove damaged condylar head/neck). In addition to providing better stabilization, the notches also provide an avenue for load transfer between the prosthesis and host bone. This will reduce forces and resultant stress experienced by fixation screws which act as the only mode of load transfer between most TMJ prostheses, especially for the condylar devices in which the collar of condylar prosthesis does not adequately contact the host bone or the medial surface of the implant does not adapt accurately to the complex geometry of patient’s mandible. Though having both medial and superior notches in the condylar prosthesis is likely to be advantageous from biomechanical viewpoint, this may make surgical implantation of the device more challenging for the surgeon. Therefore, it will be the surgeon’s choice to have either one or both notches for condylar implant.

#### 2.1.3. Design of Fossa Prosthesis

**Figure 13:**
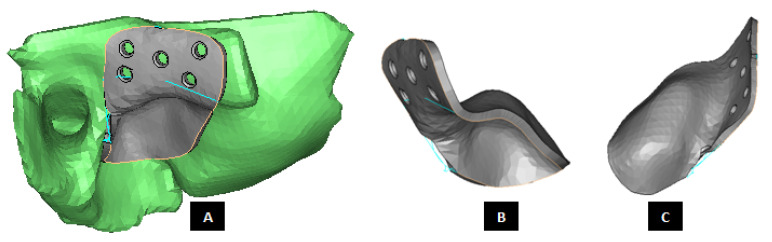
A simple custom-design of the fossa prosthesis with screw holes. (**A**) Demonstrates that the implant is designed for optimal usage of natural fossa eminence for fixation using screws. (**B**,**C**) Show different views of the implant illustrating the custom shape accurately conforms to the contours of host anatomy. The implant has constant thickness throughout its body, and the shape of articulating surface is same as that of the natural articular surface.

**Figure 14:**
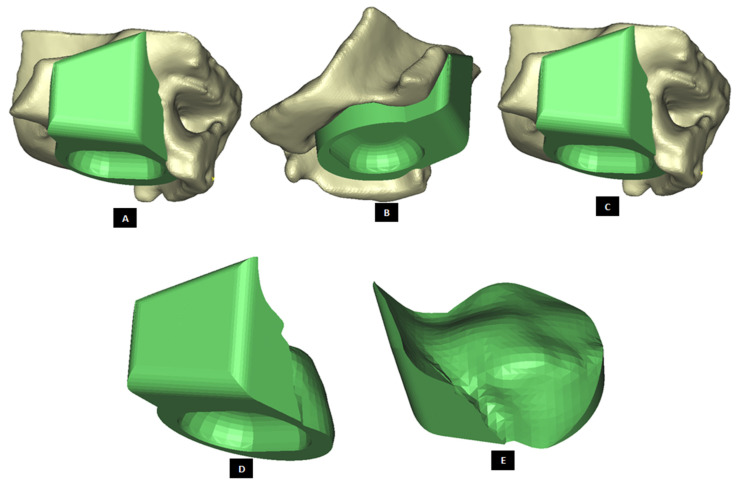
Patient-fitted design of a fossa prosthesis. (**A**–**C**) Illustrate accurate fit of the device to the patient’s natural fossa and eminence. The rectangular slot (with curved anterior and posterior edges) in inferior surface of the implant is designed to provide sufficient rotation and opportunity for anterior-posterior and medio–lateral translation of the matching prosthetic condylar head. The articular grove is designed such that it would prevent dislocation of the prosthetic condylar head during functional movements of the jaw. Visuals in (**D**,**E**) show that the superior surface of the implant is designed to accurately match the shape of natural fossa. Sufficient thickness is maintained for the lateral portion of implant to pre-drill screw holes which can host locking screws for better fixation and stability.

**Figure 15:**
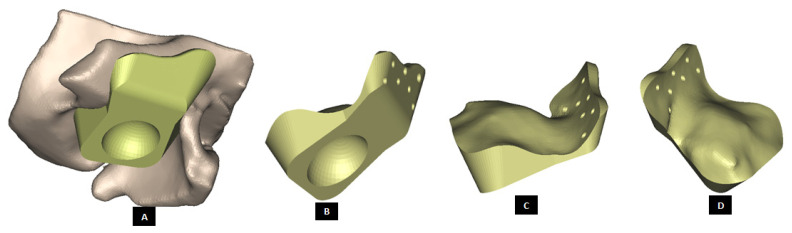
Patient-specific design of fossa prosthesis. Inferior rectangular surface of the device has a circular grove for articulation with condylar head (**A**,**B**). Visuals illustrate customized size and shape of the device for accurate fit and fixation (**C**,**D**) to native anatomical structure. Superior edge of the lateral surface (which hosts screw holes) is custom cut to follow the curvature of native eminence and bone situation.

**Figure 16:**
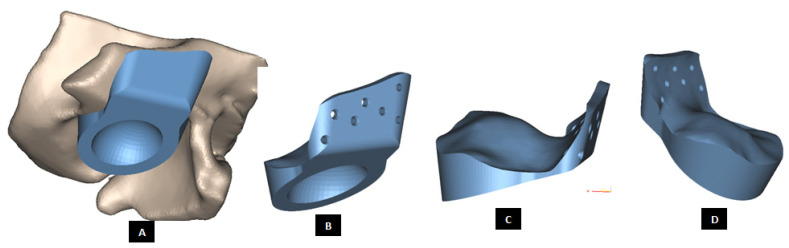
Patient-fitted fossa implant with circular inferior surface which also has a circular grove for articulation with condylar head. Visuals in (**A**–**D**) demonstrate the customized size and shape of the implant.

**Figure 17:**
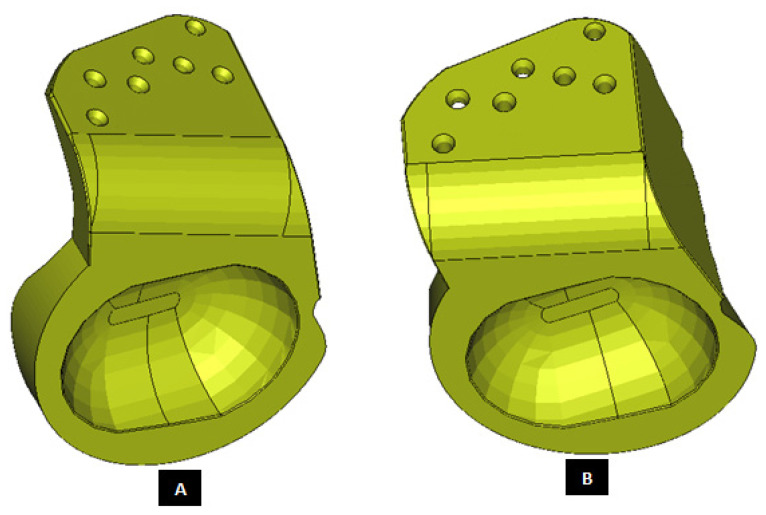
Patient-specific design of fossa prosthesis (**A**,**B**). The device has a rectangular grove (with curved anterior and posterior edges) in its inferior surface for articulation with condylar head. The uniquely designed articulating surface/hole is slanted in anterior direction. This anterior slope of articulating surface is intended to provide opportunity for anterior translation of the condylar head during movements of mandible. This feature of our fossa prostheses provides an advantage over currently available total TMJ implants which, when implanted, only rotate but do not translate during functional movements of the patient’s jaw [19].

**Figure 18:**
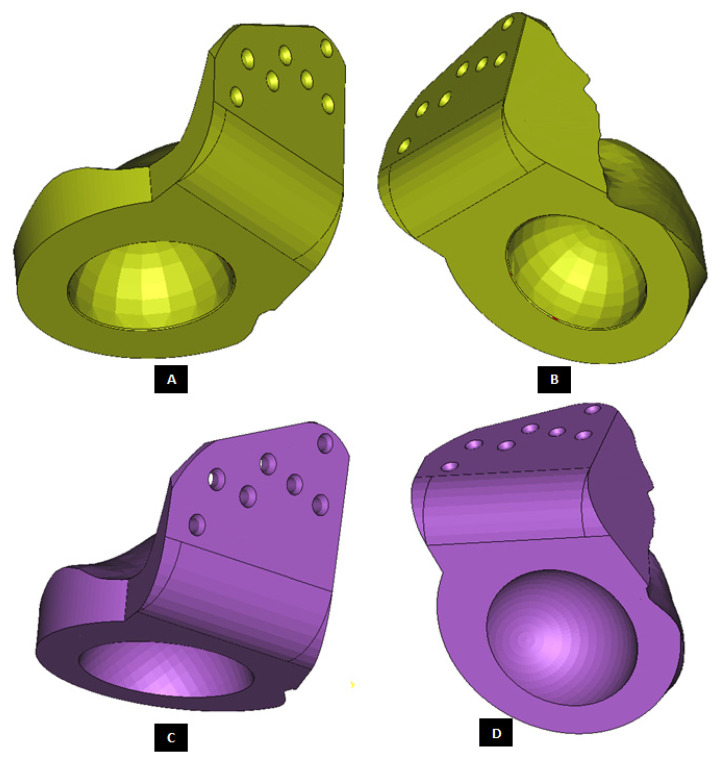
Custom-designed fossa prosthesis with circular articular surface. The device shown in (**A**,**B**) has relatively smaller articulating circular hole compared to the one shown in (**C**,**D**). Additionally, articulating surface of the device shown in (**C**,**D**) is slanted anteriorly to augment anterior translation of condylar head during mastication.

**Figure 19:**
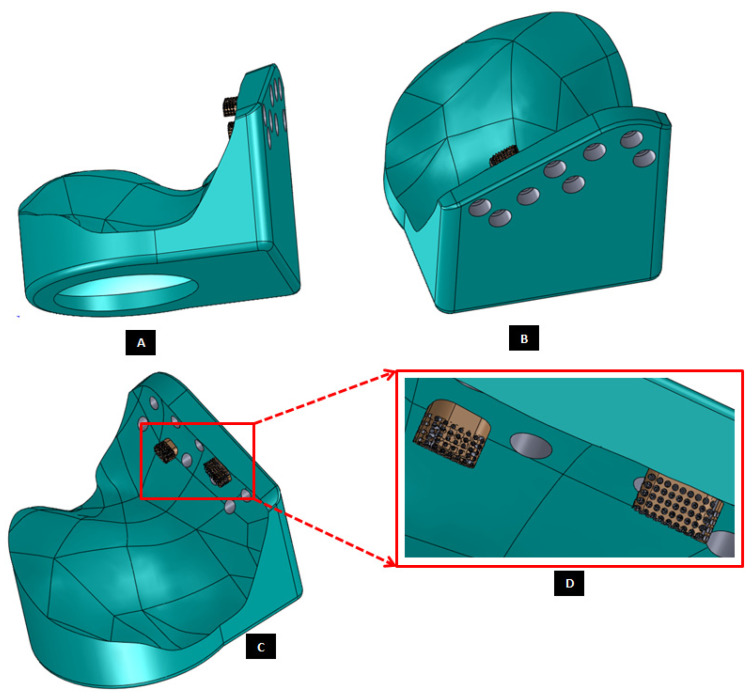
Patient-specific design of a fossa implant with circular articular surface/hole in the inferior face of the device (**A**). The device has a novel feature; perforated medial notches (**B**) protruding into host bone at implantation. Each perforated notch is designed to fit into surgically created mating grove in the host bone, thereby maximizing device stability by allowing ingrowth of bone into the prosthesis after implantation (**C**,**D**). The notches also provide a mode for load transfer between the prosthesis and native bone, thereby reducing the amount of load and resultant stress acting on the fixation screws. The surgeons can be provided with custom-designed templates and cutting guides to accurately cut the slots in native bone to accommodate perforated notches.

**Figure 20:**
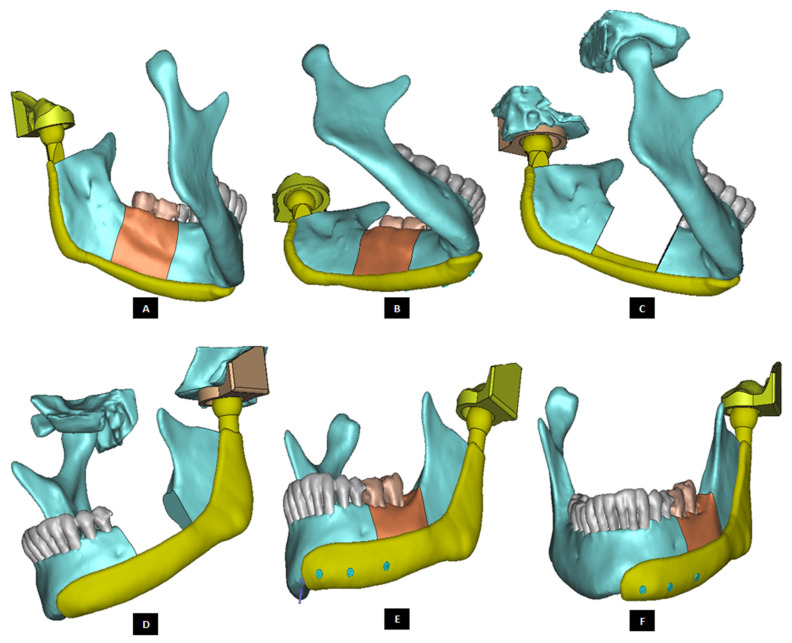
Shape outline of the patient-specific total TMJ prosthesis. Ramal component of the prosthesis is extended anteriorly up to the chin (**A**–**F**) to support mandibular host bone and graft (with aesthetic dental implant) after removal of the imaginary tumor (shown in red) in the left mandibular body/molar region.

**Figure 21:**
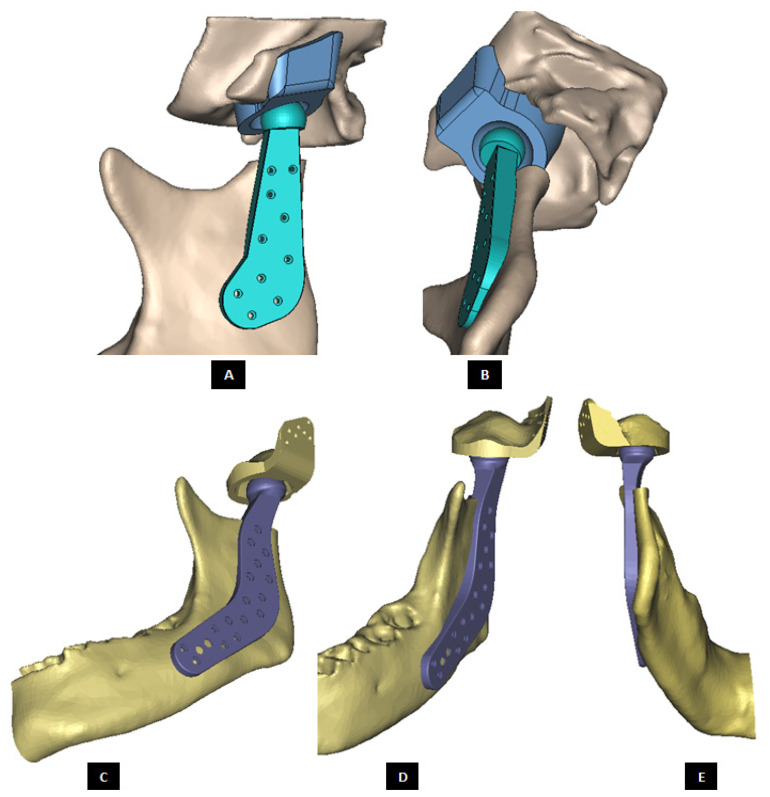
Patient-specific total TMJ prostheses with different articulations and fixation (**A**–**E**).

Fitting the skull is a major problem in TMJ reconstruction patients because of the irregular shape of their TMJs [14,15] The patient-specific design approach enables developing accurately fitting models for the complex shape of patient’s fossa-eminence anatomy. Using a similar design approach discussed earlier for the condylar implants, patient-fitted custom designs of fossa prosthesis can be developed such that the device fits accurately to the available host bone. Such custom designed fossa implants can correctly adapt to the natural components of patient’s TMJ, and provide improved stability through locking screws and perforated notches fitting into patient’s skull. Figure 13, Figure 14, Figure 15, Figure 16, Figure 17, Figure 18, Figure 19, Figure 20 and Figure 21 show different shapes and features of our custom-designed fossa prostheses.

#### 2.1.4. Design of Screws

Unlike hip or knee joints, the bony anatomy of mandibular ramus and temporal glenoid fossa do not afford the use of modular stock components for TMJ TJR that can be stabilized initially with press-fitting or cementation [2]. Therefore, TMJ devices have to rely only on screws for initial fixation and stabilization of their components. Clinicians have underlined the need for improved methods of internal fixation of prosthetic TMJ devices to minimize or eliminate implant loosening and joint failure [1]. The position of inserted screws was more important than the number of screws for stable fixation of the condylar TMJ prosthesis [20]. Our methodology of designing patient-specific total TMJ prostheses based on anatomically accurate 3D models provides realistic and accurate options in deciding positions of fixation screws for the prostheses. The positions of pre-drilled screw holes in the prostheses can be selected to avoid unintentional injury to delicate structures in the vicinity while ensuring stable fixation of the devices. Moreover, unlike stock TMJ implants, the custom-designed TMJ prostheses do not have any unused screw holes which may act as stress-risers under functional in-vivo loading post-implantation.

**Figure 22:**
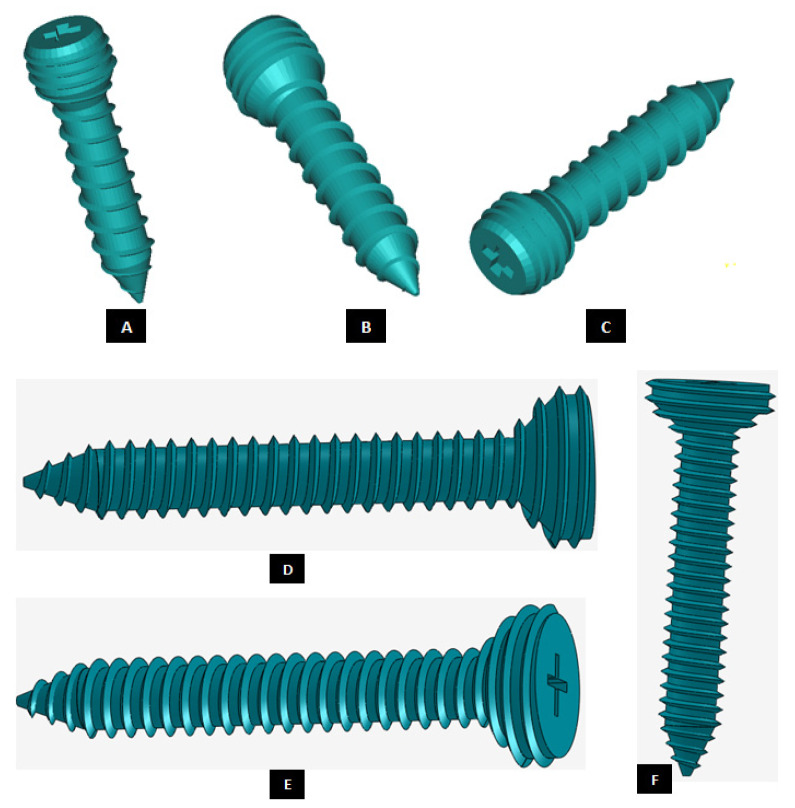
Custom-designed screws with locking mechanism. The threads on the screw-head surface provide improved/optimal fixation by firmly engaging in the matching threads in the screws holes of either condylar/ramal or fossa component of the total TMJ prosthesis (**A**–**F**).

**Figure 23:**
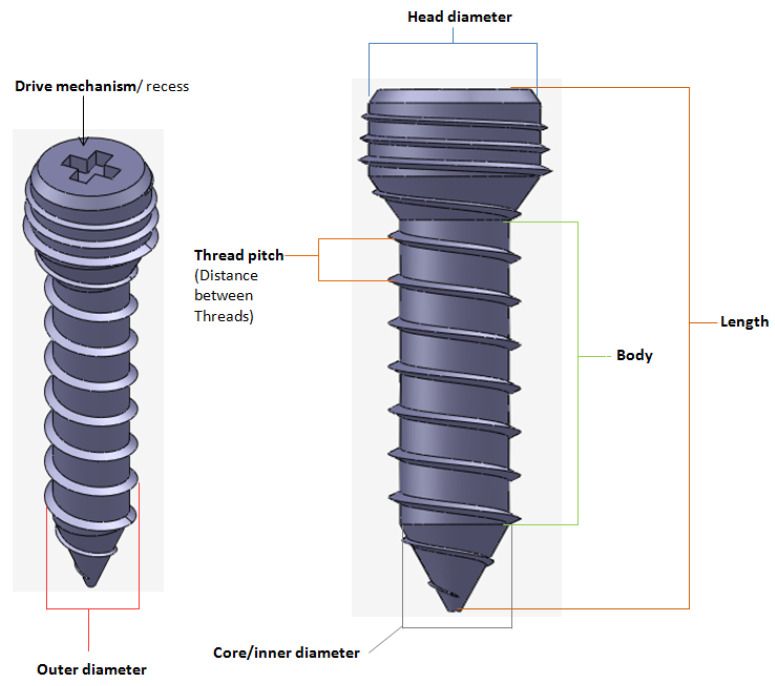
A custom-designed locking screw for TMJ prosthesis. Visuals show different features of the screw. Total length of the screw depends on the size of prosthesis and native bone. The body/shaft of screw is designed long enough to utilize maximum amount of host bone (condyle/ramus or fossa eminence) but avoid protrusion of screws from medial surface of the bone. Length of the screw head varies depending on the thickness of condylar or fossa prosthesis in the particular screw-hole location. The outer diameter of screw is kept in the range of 1.5 mm–3.00 mm as this range is reported to be optimal for the screws of TMJ implants. The screw has varying pitch, with more threads per unit length of screw-head than the body/shaft.

Motion of implanted TMJ prosthesis under load can cause degeneration of the surrounding bone, which may lead to further device loosening and possible failure [1]. Screws may loosen with time and function, requiring replacement. For long-term success of the TMJ implants, forces from the implant to the bone and vice versa must occur without relative motion or without intermittent loading [2,21]. The use of bone screws with sharp threads in TMJ implants prevents movement between the screw head and prosthesis [15]. The custom-designed screws of our TMJ prostheses system provide optimal fixation through locking mechanism—a unique feature not currently offered by any of the US FDA-approved TMJ TJR devices (see Figure 22 and Figure 23). Threads on the screw-head surface provide high grade fixation by firmly engaging with the matching threads in the screw hole of either condylar/ramal or fossa implants. The possibility of movement between the screws and prosthesis can be eliminated by using such locked screws. In addition to the surgical condylectomy guides, our methodology also provides the surgeons with customized screw-drill-guides and templates for the TMJ prostheses. These drill guides are intended to create a hole of preferred dimension (diameter and depth), and at the accurate location and orientation for each screw as prescribed by the surgeon.

#### 2.1.5. Implant Materials

Using advantageous physical characteristics of biocompatible materials is an essential aspect in the design and manufacture of a successful prosthetic device. Some of the important characteristics of materials used to manufacture the TMJ prostheses from are biocompatibility, mechanical strength, low wear-rate, and harmless wear particles. Advancement in materials research has led to materials [22] such as medical grade pure titanium (Ti), titanium alloy (Ti-6Al-4V), cobalt-chromium-molybdenum (Co-Cr-Mo), and ultrahigh molecular weight polyethylene (UHMWPE) becoming gold standard for low friction orthopaedic joint replacement, Table 3.

Wrought Co-Cr-Mo is reported to have excellent wear resistance when articulated against UHMWPE in the non-movable articulating surface of most orthopaedic TJR devices [23]. However, TMJ is a highly mobile joint in which articulating surfaces of the reconstructed joint undergo repeated mechanical stresses resulting from movement of the jaw. Metallurgical flaws, such as porosity, found in cast Cr-Co are suggested to cause the fatigue failure of Cr-Co TJR components resulting in noxious metallic debris in the patient’s body [23].

Titanium alloy (Ti-6Al-4V) for condylar/ramus component and bone screws, and UHMPE for fossa component is based on their favorable characteristics and successful long-term applications reported in scientific and clinical literature. Unalloyed titanium reacts rapidly with oxygen in the air to form a thin (<10 μm) layer of chemically inert titanium oxide which provides a favorable surface for biointegration of prosthesis with bone [2]. In addition to its biocompatibility and biointegration, Titanium also offers properties of strength, corrosion resistance, ductility, and machinability [23]. UHMWPE is a linear unbranched polyethylene chain with a molecular weight of more than one million. UHMWPE is shown to have excellent wear and fatigue resistance for a polymeric material [24]. Untill year 2011, no cases of UHMWPE particulation-related osteolysis have been reported in the TMJ prostheses literature [2,25].

### 2.2. FEA of Total TMJ Implant

**Figure 24:**
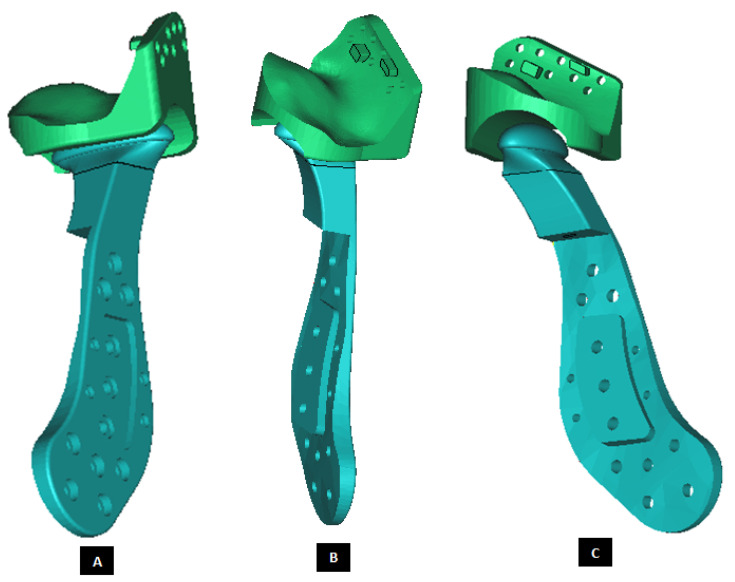
A patient-specific total TMJ prosthesis with medial notches in fossa and condylar components. (**A**) Shows anterior–lateral view of the ‘notched implants’ with screw holes. Fossa prosthesis has two medial notches to be fit into host bone (**B**,**C**). The articular surface of fossa implant has medio–lateral openings, and is designed to allow optimal anterior and medial translation along with rotation of the prosthetic condylar head along the medio–lateral axis.

**Figure 25:**
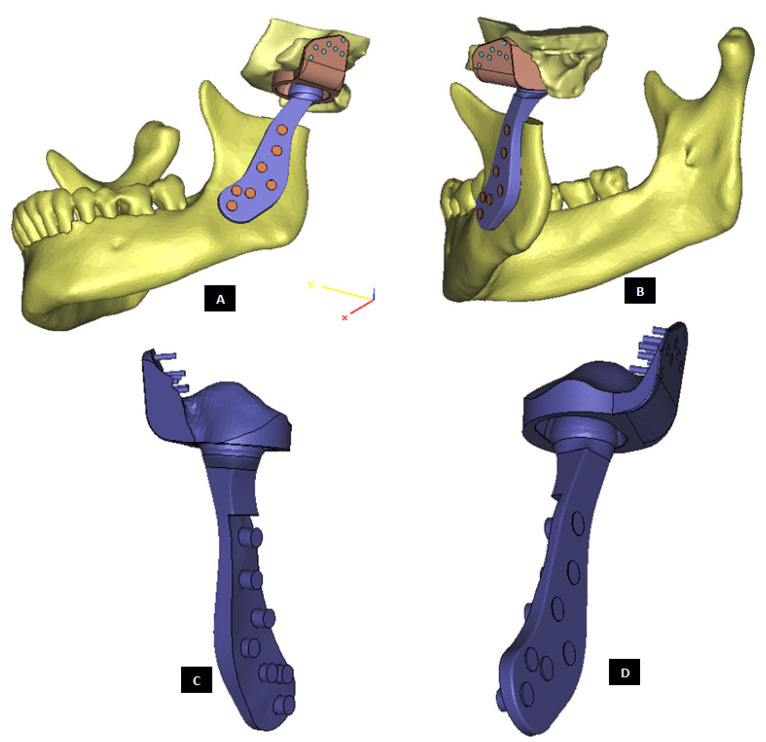
A patient-specific total TMJ prosthesis. (**A**,**B**) Show two views of the ‘simple’ total TMJ prosthesis along with left fossa bone and mandible after removal of left condyle. (**C**,**D**) Show two views of the total TMJ along with screws.

Methods for biomechanical assessment of prosthetic TMJ must be developed to make the implantation outcomes more predictable and reliable, and to evaluate the methods of device fixation to minimize or eliminate implant loosening and joint failure [1]. We performed FE simulations of two patient-specific total TMJ prostheses—one device with medial notches fitting into the groves created in the host bone (see Figure 24) and another ‘simple implant’ without such notches in the condylar and fossa components (see Figure 25)—using our validated methodology described elsewhere [18]. The objective of this study was to investigate stress and strain distribution in prosthetic components and host bone surrounding the screws under normal and worst-case/over–loading conditions. To account for the user-induced errors due to variations in selecting the nodes of FE mesh for applying boundary conditions and loads, we performed three repetitions of FE simulation under each loading condition for both total TMJ prostheses systems. Results reported in Table 4 and Table 5 are average of the values obtained from three runs of each FE simulation.

#### 2.2.1. FE Modeling and Mesh Generation

**Figure 26:**
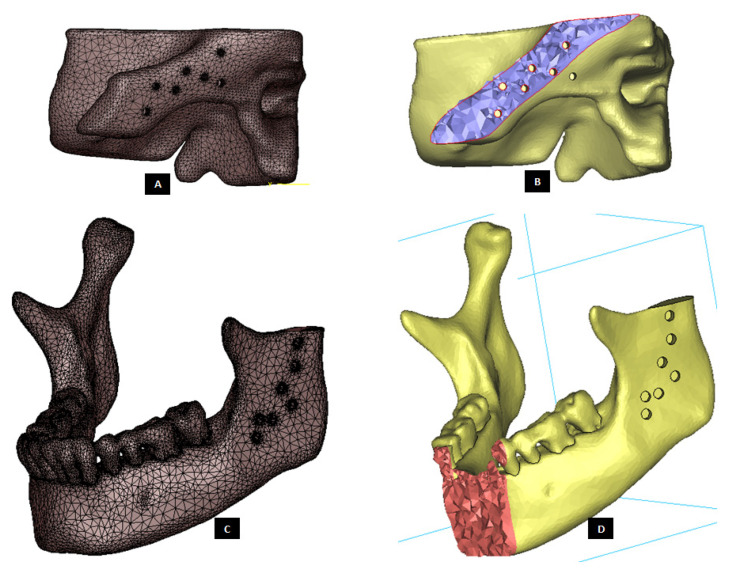
Three-D finite element mesh of the host bone components prepared for total prosthetic replacement of the left TMJ. (**A**) Shows FE surface mesh of left fossa, and (**B**) shows a lateral cross-section of the 3D volume mesh of left fossa bone with screw holes. Similarly, (**C**,**D**) show surface mesh and anterior cross-section of volume mesh, respectively, of the mandible with screw holes and removal of damaged left condyle.

**Figure 27:**
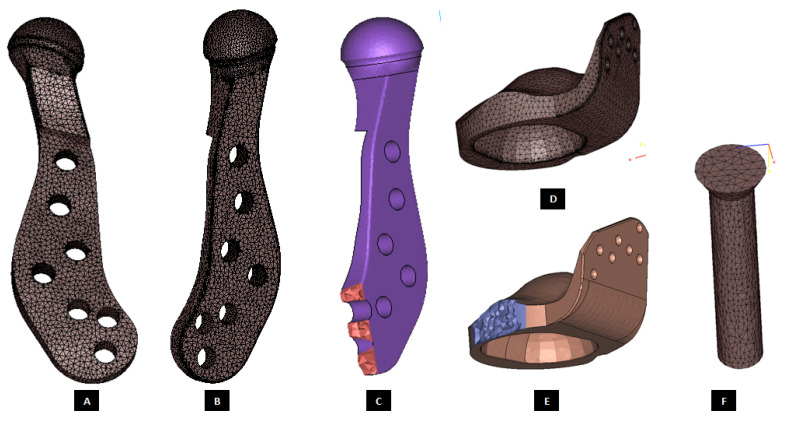
Three-D finite element mesh of the components of patient-specific total TMJ prostheses. (**A**–**C**) Show FE mesh of the condylar/ramal component of the ‘simple’ TMJ implant (without notches). (**D**,**E**) Show FE mesh of the fossa component, and (**F**) demonstrates FE mesh of a screw for device fixation.

Subject-specific 3D anatomical reconstruction of patient’s mandible and skull/articular eminence was performed using commercial software Mimics v14.12 (Materialise, Plymouth, MI, USA) from computed tomography (CT) scans of patient’s TMJ. Upon importing the patients CT images in Mimics, anatomical model comprising of patient’s mandible and fossa eminence was developed from the CT scan by performing a series of operations such as image processing, segmentation, mask formation for bone and teeth, region growing, and calculation of 3D equivalent similar to the 3D reconstruction method described elsewhere [18]. Using the design methodology discussed in previous sections, two patient-specific total TMJ prostheses systems—a ‘simple implant’ without notches, and another ‘implant with notches’—were designed for total reconstruction of the patient’s left TMJ (see Figure 24 and Figure 25). For FE simulations, volume bound within surfaces of anatomical components (cortical bone, cancellous bone, and teeth) and prosthetic components (condyle, fossa, and screws) were meshed. Three-D volume mesh was generated for each of these components with ten-node quadratic tetrahedral elements of type C3D10 (see Figure 26 and Figure 27). Mesh convergence was achieved using the technique discussed previously [18]. The finite element analyses were performed using a commercial FE package ABAQUS v6.10 (SIMULIA, Providence, RI, USA).

#### 2.2.2. Model Constraints and Loads

**Figure 28:**
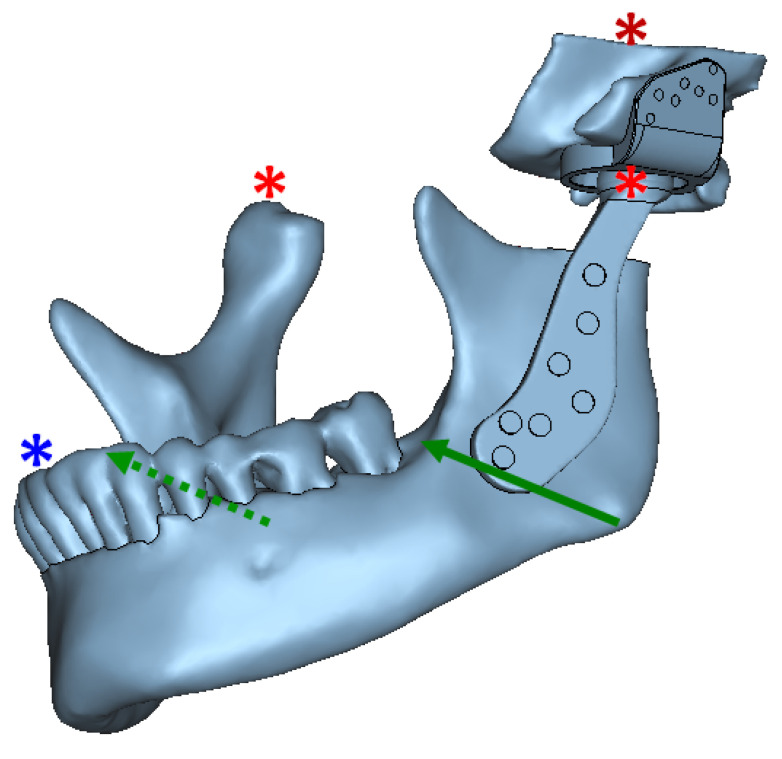
Assembly of all parts of the FE model (including anatomic and prosthetic components), and schematic representation of model constraints and load application for FE simulation of total TMJ prostheses and anatomical components. Green arrows depict the location and direction of bite forces applied in the angulus region on both sides of the mandibular mesh. The asterisks indicate constrained nodes at condyle, fossa, and incisor teeth. Left prosthetic condylar head and right natural condylar head were constrained such that they could only rotate along the medio–lateral axis and translate in anterior-posterior direction. The nodes at incisor teeth were so constrained such that they could only rotate. The entire fossa host bone was constrained in all directions. The interface between prosthetic condylar head and articulating surface of prosthetic fossa was modeled as sliding contact. The prosthesis-to-bone, screw-to-prosthesis, and screw-to-bone interfaces were assumed to be bonded. The interfacial and boundary conditions were kept similar for normal and over–load configurations; and only magnitude of applied forces was changed across the two loading configurations.

As illustrated in Figure 28, the condylar head of the prosthetic TMJ was allowed to rotate along the medio–lateral axis, and translate in anterior-posterior direction. Similarly, for the TMJ on contralateral side, the natural condylar head was allowed to only rotate along the medio–lateral axis, and translate in anterior-posterior direction. The incisor teeth were fixed so that they could not translate in three directions, but could rotate. The entire fossa host bone was constrained in all directions.

The interface between prosthetic condylar head and articulating surface of fossa prosthesis was modeled as sliding contact with a coefficient of friction of 0.3. The interface between the prostheses and bone in contact was modeled with contact elements having a coefficient of friction of 0.42 as reported in literature [26]. The screw-to-prosthesis and screw-to-bone interfacial conditions were assumed to be bonded. Since use of locking screws eliminates the possibility of movement between screw and prosthesis, the interface condition between screw heads and TMJ prostheses (condylar and fossa) was assumed to be perfect bonding. Two oblique bite forces, each 200 N for normal loading condition and 400 N for over–loading/worst-case scenario, were applied to the mandibular model in the angulus area as shown in Figure 28.

#### 2.2.3. Material Properties

Young’s modulus and Poissson’s ratio of anatomical components (fossa and mandible bone with teeth), titanium alloy (for condylar component and all screws), and UHMWPE (for fossa component) were selected as listed in Table 3. All anatomical components of the model (i.e., cortical bone, cancellous bone, and teeth) were assigned properties of the cortical bone similar to other researchers [27,28] who have previously followed this practice since variation in material properties of these components have negligible influence on biomechanics of the mandible. All materials used in FE models were assumed to be isotropic, homogeneous, and linearly elastic [26]. Static FE simulations were performed using ABAQUS software. Three repetitions/runs of FE simulation under each loading condition were performed for both types of total TMJ implants to account for any user-induced errors such as variation in selecting exactly the same nodes of FE mesh across different simulations. The results summarized in Table 4 and Table 5 are average of three simulations for each loading condition for both types of total TMJ prosthesis.

**Table 3:** Material properties for anatomical and prosthetic TMJ components.

Part	Young’s Modulus (MPa)	Poisson’s Ratio	References
Host bone	1.47 × 10^4^	0.3	[25,29]
Titanium alloy (Ti-6Al-4V)	1.10 × 10^5^	0.3	[28]
UHMWPE	830	0.317	[30]

**Table 4:** Peak von Mises stresses developed in condyle/ramus and fossa components, and fixation screws of the simple and notched designs of patient-specific total TMJ prostheses during FE simulations under normal and worst-case/over–loading configurations.

Implant Type	Loading Type	Peak von Mises Stress in Implant Components (MPa) *			
		Condyle/Ramus	Condylar Screws	Fossa	Fossa Screws
Simple	Normal	44.3	61.4	11.3	28
	Over–load	56.7	106.7	14.2	43.1
With Notches	Normal	42.6	59.6	10.5	23.4
	Over–load	59.1	108.3	13.4	38.6

* Average of three simulations performed under similar constraints and loading at three different times (to account for variations induced by the user/operator).

**Table 5:** Peak stress and strain developed in host bone surrounding the fixation screws of the simple and notched designs of patient-specific total TMJ prostheses during FE simulations under normal and worst-case/over-loading configurations.

Implant Type	Loading Type	Peak von Mises Stress in Host Bone Adjacent to Screw Holes (MPa) *		Peak von Mises Strain in Host Bone Adjacent to Screw Holes (µStrain) *	
		Condyle/Ramus	Fossa	Condyle/Ramus	Fossa
Simple	Normal	4.7	3.5	1983	1253
	Over–load	13.6	7.4	3586	1711
With Notches	Normal	4.3	3.2	1893	1210
	Over–load	12.5	7.1	3374	1564

* Average of three simulations performed under similar constraints and loading at three different times (to account for variations induced by the user/operator).

## 3. Results

**Figure 29:**
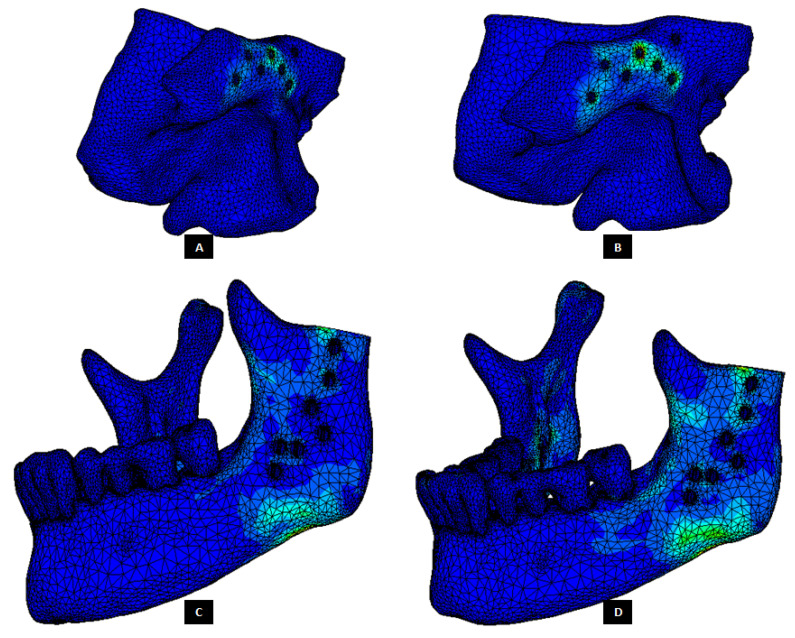
Stress distribution in the host bone components during FE simulations of the total TMJ replacement with custom designed simple TMJ prostheses. (**A**,**B**) Show von Mises stress in the fossa bone under normal and worst-case/over–load configurations, respectively. (**C**,**D**) Show von Mises stress in the mandibular bone under normal and worst-case/over–load configurations, respectively.

**Figure 30:**
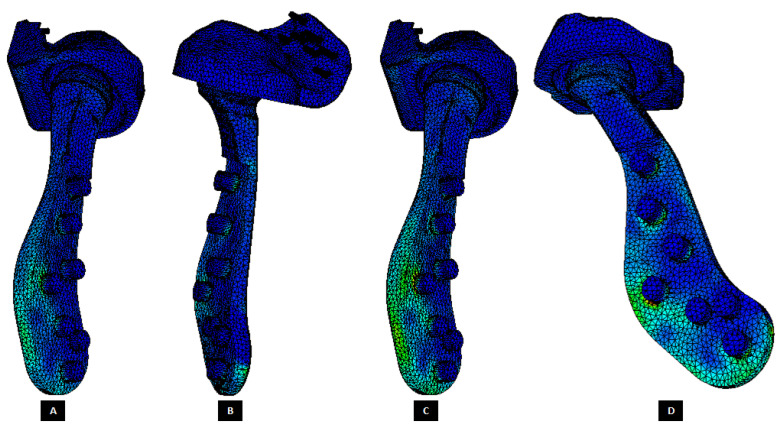
Peak von Mises stress in patient-specific ‘simple’ TMJ prosthesis (without notches) during FE simulations of two different loading scenarios. (**A**,**B**) Show von Mises total TMJ prosthesis under normal loading configuration. (**C**,**D**) Show von Mises stress profile in the prosthesis during FE simulation of worst-case/over-loading scenario.

**Figure 31:**
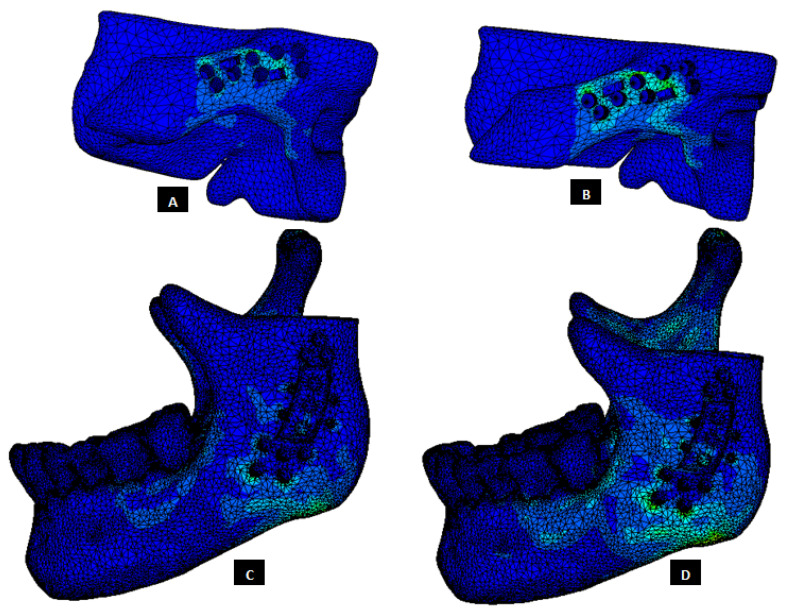
Stress distribution in the host bone components during FE simulations of the total TMJ replacement with custom designed TMJ prostheses with medial notches. (**A**,**B**) Show von Mises stress in the fossa bone under normal and worst-case/over-load configurations, respectively. (**C**,**D**) Show von Mises stress in the mandibular bone under normal and worst-case/over-load configurations, respectively.

**Figure 32:**
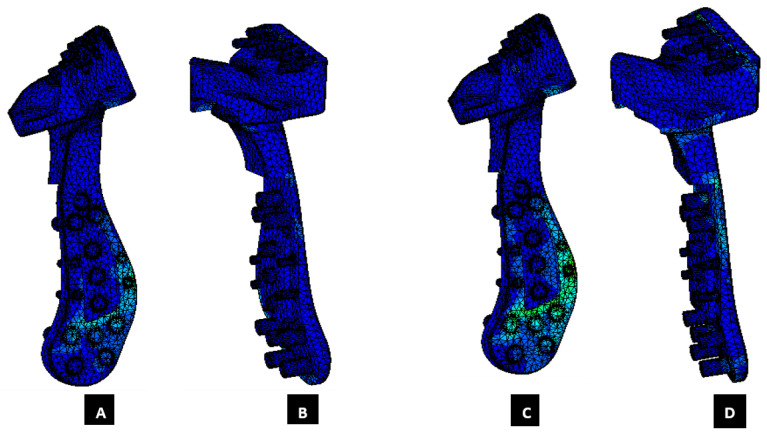
Peak von Mises stress in patient-specific ‘notched’ TMJ prosthesis (with medial notches) during FE simulations of two different loading scenarios. (**A**,**B**) Show von Mises total TMJ prosthesis under normal loading configuration. (**C**,**D**) Show von Mises stress profile in the prosthesis during FE simulation of worst-case/over-loading scenario.

The von Mises stress and micro-strain in the TMJ prostheses (fossa and condylar), screws, and native bone in regions adjacent to screws were measured. Figure 29 and Figure 30 show visuals of stress profiles in the anatomical components and simple TMJ prostheses, respectively. Figure 31 and Figure 32 show visuals of stress profiles in the corresponding anatomical components and ‘notched’ TMJ prostheses, respectively. Table 4 summarizes peak von Mises stress occurred in prosthetic components and screws. Peak von Mises stress and strain developed in host bone surrounding the fixation screws of simple and notched TMJ prostheses under normal and worst-case/over-loading configuration are summarized in Table 5.

### 3.1. Stress and Strain in Native Bone

Small difference in the stress and micro-strain occurred in the host bone adjacent to screws in condylar and fossa components of both total TMJ prosthesis systems. For both types of implant designs, von Mises stress in the bone surrounding fixation screws was in the range of 3.2–4.7 MPa and 7.1–13.6 MPa under normal loading and over–loading, respectively (see Table 5, Figure 29 and Figure 31). These results are comparable to the findings reported by [31] who studied stress distribution in the screws of a condylar implant and host bone. von Mises strain in the bone surrounding prosthetic screws ranged from 1210 microstrain to 1983 microstrain during normal loading, and from 1564 microstrain to 3586 microstrain during over-loading condition. A strain higher than 4000 microstrain can cause hypotrophy of bone [32]. The highest micro-strain in host bone in this study is below the hypertrophy limit. Moreover, use of more screws at appropriate locations would further lower the chances of higher strains capable of bone formation around the screws.

### 3.2. Stress and Strain in TMJ Implants

**Figure 33:**
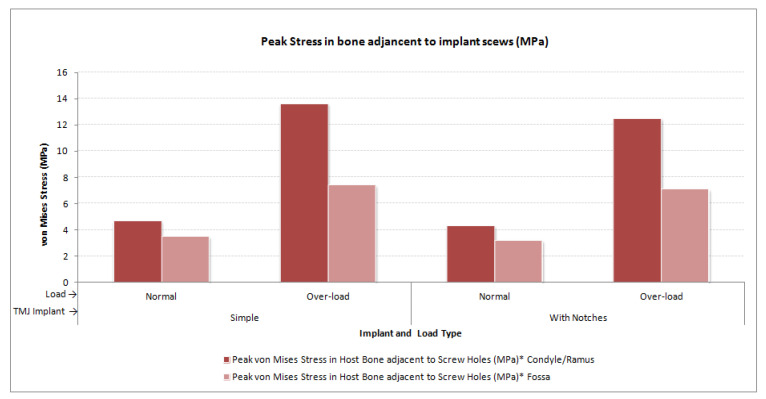
Peak von Mises stress in the mandibular and fossa bone adjacent to fixation screws of total TMJ prostheses during FE simulations under normal and worst-case/over-load configurations.

**Figure 34:**
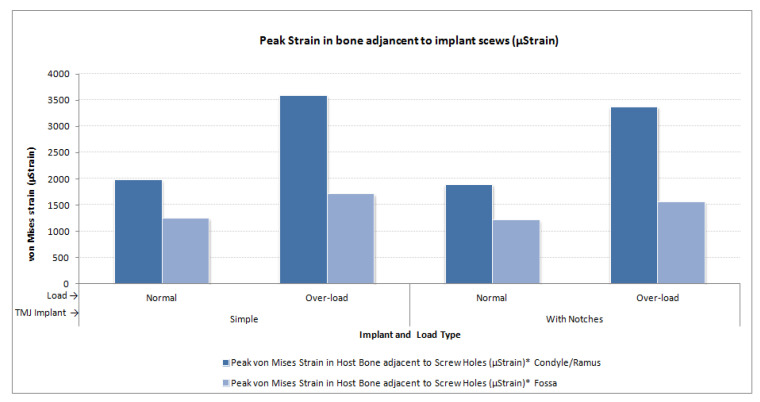
Peak micro-strain in the mandibular and fossa bone adjacent to fixation screws of total TMJ prostheses during FE simulations under normal and worst-case/over-load configurations.

FE simulations resulted in lower stress in anterior part of the condylar and fossa prostheses (see Figure 30 and Figure 32) similar to what found in [20,28] although their FE studies included only the condylar TMJ implants with different loading conditions. The von Mises stress found in condylar and fossa components of both types of implants were lower than the yield strength of their materials Figure 33, Ti-6Al-4V and UHMWPE, respectively. The trends in the stress and strain profiles under normal and over-loading conditions were similar in both types of total TMJ prostheses. von Mises stresses of higher magnitude were developed in condylar neck, posterior part of condylar head, and inferior region of ramal component compared with rest of the condylar/ramus prosthesis. For fossa component, magnitude of von Mises stress and strain was higher in the posterior region on the articulating surface, Figure 33 and Figure 34.

**Figure 35:**
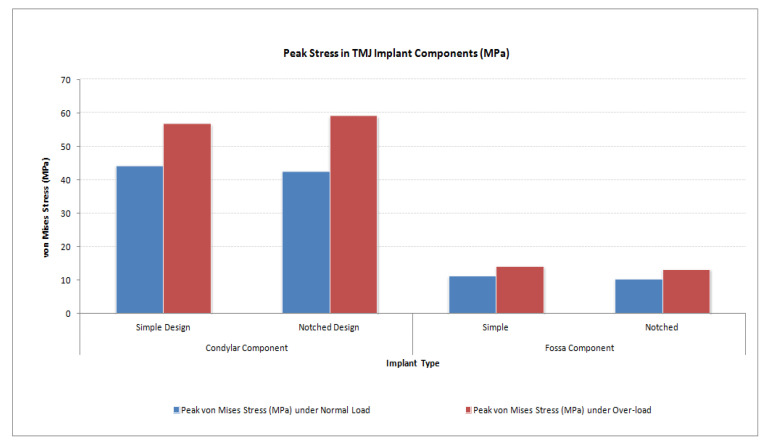
Peak von Mises stress in condylar/ramus and fossa components of patient-specific total TMJ prostheses during FE simulations under normal and worst-case/over-load configurations.

Other than where the actual loads and constraints were applied, the stress concentration was highest around the inserted screw and the screw holes in the host bone. The medial notches in the condylar and fossa prostheses are designed to provide improved stability by promoting post-implantation bone growth into the perforated surfaces of the notches. Von Mises stress in the notch regions of the condylar and fossa implants were less than that in the screw regions, indicating that the notches may not act as stress risers in the device. Stress profile in the host bone portion where the medical notches of the implant are inserted show stresses lower than that at the screw holes, but higher than those in other parts of the host bone. This indicates that the stress developed in the notches under functional loading may augment bone growth into the perforated notches, thereby maximizing the opportunity for improved stability of the prostheses. Also, in all simulations, the peak von Mises stresses in the condylar component were higher than those in the fossa component of the total TMJ prostheses (see Figure 35). This may have resulted from the model constraints which allowed mobility of the condylar/ramal prosthesis along with natural mandible and kept the artificial fossa fixed in its position along with the host fossa bone.

### 3.3. Stress and Strain in Screws

**Figure 36:**
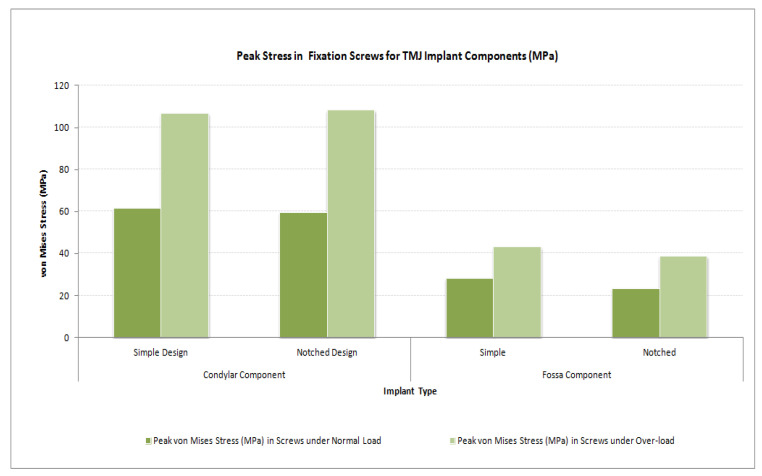
Peak von Mises stress in the fixation screws for condylar/ramus and fossa components of patient-specific total TMJ prostheses during FE simulations under normal and worst-case/over-load configurations.

Peak stress and strain the implant fixation screws are summarized in Table 4 and Figure 36. In fixation screws for condylar and fossa components, the highest magnitude of stress values occurred at the neck portion of screws. However, the highest stresses in all screws were found to be less than the ultimate stress as well as yield point of the screw material (Ti-6Al-4V). This trend of stress profile in screws is similar to that reported in [31] who studied stress distribution in a stock condylar prosthesis and screws using FE method. The highest von Mises stresses found in screws in the present study are much lower than those reported in [31]. This discrepancy suggests that screws used for fixation of the condylar component of patientspecific total TMJ prostheses undergo lower load and resultant stress while transferring the functional loads between implant and host bone. This further suggests that the custom-designed implants offer better adaptation to the host bone (compared to their stock counterparts) and partly transfer the load directly to the bone in contact (e.g., at the location where condylar collar of the implant sits superiorly on natural ramus after condylectomy), thereby reducing the exposure of screws to higher loads and stresses.

Among the screws, the highest stresses occurred in the neck portion of two condylar screws—one placed most inferiorly, and another placed most posteriorly and at the curvature of the ramal part of the implant. This is contradictory to what found in [31,33] reporting highest stresses in the condylar screw placed most superiorly (near the neck of implant). However, both these studies included a stock condylar TMJ implant in which the implant collar did not contact or adapt to the host bone as it does in the present study. Also, these researchers applied a vertically downward force at the top of prosthetic condylar head whereas we applied load in mandibular angle region.

Screws used for both condylar and fossa components showed von Mises stresses of higher order at their interfaces with prostheses, especially in the region of screw neck and at the site of prosthesis-bone junction. This may have happened as the screws carried more load when they served as a medium of load-sharing between the prosthesis and host bone. As listed in Table 4, maximum von Mises stress generated in the screws was relatively higher than that in the corresponding prosthetic component these screws were used for fixation of.

## 4. Discussion and Conclusions

In view of scarcity of published literature about design methods and biomechanical analysis of total TMJ implants, the present study provides good reference work for patient-specific design and biomechanical evaluation of such designs through FE simulations. Few published studies have investigated biomechanics of the artificial TMJ implants. In our knowledge, no study has reported FE analysis of total TMJ prostheses. The present study can serve as a reference for the clinicians regarding advantageous features of the patient-specific total TMJ implants. Moreover, design methodology and FE findings of this study can provide industrial designers with reference data for improving their products, especially the custom-designed products intended for patients with complex and challenging anatomic situations.

Limitations of this study must be considered when reviewing implant designs and evaluating FE results. The main focus of this study was the patient-specific design and biomechanical analysis of the total TMJ prostheses. The skill of the surgical approach (preauricular incision or retromandibular incision) and patient-related issues such as long-term effects were not considered. Therefore, the surgeons should be careful when applying the findings from this study to clinical situation. The present study used only one set of material properties for patient’s host bone. Future investigations should assess effect of altered bone quality on the performance of total TMJ replacement. Also, material properties of bone were assumed to be homogeneous and isotropic. Although this represents a major simplification, other studies have demonstrated that this is acceptable [20,26,34]. Only two loading conditions (with loads applied at the mandibular angle) were used in this study. Other muscle forces which are normally present would also affect the mandibular biomechanics. Although several studies have suggested that forces from other muscles do not exert major effects in the mandible [20,26,35], future work should consider using more sophisticated FE models. It will be beneficial to evaluate the effect of screw positions on biomechanical performance of total TMJ prostheses. Future work should also include more comprehensive non-linear and dynamic FE simulations using different implant materials.

In summary, this study demonstrates that our custom-design approach offers potential for stable and durable total TMJ reconstruction, and that the FE models can reproduce information useful in design and assessment of total TMJ prostheses. Findings of this study provide a good basis for future work focusing on developing a more refined and standardized method for custom design of total TMJ prostheses, and pre-clinical FE tests for design verification and validation.

## Figures and Tables

**Table 1:** Indications for the alloplastic reconstruction of the temporomandibular joint (TMJ).

Sr. No.	Indications for Alloplastic TMJ Reconstruction
1.	Ankylosis or reankylosis [1,3,4], degeneration, or resorption [3,4] of joints with severe anatomic abnormalities.
2.	Failed autogenous grafts in multiply operated patients [1,3,4].
3.	Destruction of autogenous graft tissue by pathology [1,3,4].
4.	Failed Proplast–Teflon that results in severe anatomic joint mutilation [1,3,4].
5.	Failed Vitek–Kent total or partial joint reconstruction [1].
6.	Severe inflammatory joint disease, such as rheumatoid arthritis which results in anatomic mutilation of the joint components and functional disability [1,3,4].

**Table 2:** Criteria for the successful alloplastic total reconstruction of the TMJ.

Sr. No.	Requirements/Criteria for Success of Alloplastic Total Joint Replacement Devices
1.	The materials from which the devices are made must be biocompatible [1,2,15,16].
2.	The devices must be designed with sufficient mechanical strength to withstand the loads delivered over the full range of function of the joint [1,2,15,16].
3.	The devices must be stable in-situ [1,2,15,16].
4.	The surgery to implant the prosthesis must be performed for the proper indications, and it must be performed aseptically [1,2,16].
5.	The prostheses should imitate the condylar translation during mouth opening, and without restricting movements of non-replaced TMJ [15].
6.	The prostheses should be fitted correctly to the mandible and the skull [15].
7.	Expected lifetime of more than 20 years [15].
8.	Low wear rate; and wear particles must be tolerated by the body [15].
9.	Simple and reliable implantation procedures [15].
